# Post COVID-19 fibrosis, an emerging complicationof SARS-CoV-2 infection

**DOI:** 10.1016/j.idcr.2020.e01041

**Published:** 2020-12-31

**Authors:** Mousa Ahmad Alhiyari, Fateen Ata, Mohd Islam Alghizzawi, Ammara Bint I Bilal, Ahmad Salih Abdulhadi, Zohaib Yousaf

**Affiliations:** a Department of Internal Medicine, Hamad General Hospital, Hamad Medical Corporation, Doha, Qatar; b Department of Radiology, Hamad General Hospital, Hamad Medical Corporation, PO BOX 3050, Doha, Qatar

**Keywords:** Coronavirus infection 2019, COVID-19, Severe acute respiratory syndrome coronavirus 2, SARS-CoV-2, Acute respiratory distress syndrome, ARDS, Pulmonary fibrosis, Dyspnea

## Abstract

SARS-COV-2 has created one of the most massive pandemics in modern history. There is a rapid accumulation of data on its epidemiology, clinical course, diagnosis, management, and complications. One of the sequelae of COVID-19 pneumonia and acute respiratory distress syndrome (ARDS) is pulmonary fibrosis. There is a dearth of accurate data on the prevalence of pulmonary fibrosis post-COVID-19. We report a patient who developed dyspnea secondary to pulmonary fibrosis after successful treatment of COVID-19 pneumonia.

## Highlights

• Lung fibrosis is an emerging complication of SARS-CoV-2 infection.
• Exertional desaturation may be present in treated COVID-19 patients at discharge.
• A 6-minute walk test may be of prognostic value on COVID patients at discharge.

## Introduction

Severe acute respiratory syndrome coronavirus 2 (SARS-CoV-2), the virus behind the most massive pandemic of the decade, has resulted in 33 million infections and nearly 1 million deaths globally as of September 2020. The pandemic currently has a variable epidemiologic course, with a rise in the number of new cases in some countries and a decline in others [1]. Some countries are currently experiencing a second wave of infection. There is a lot of research ongoing to understand the pathophysiology, clinical course, and management of COVID-19 infection, with a particular focus on treatment modalities.

One of the complications of COVID-19 pneumonia and ARDS is pulmonary fibrosis [2]. Although there is currently no clinical data on the frequency and mechanism of post-COVID-19 pulmonary fibrosis, it is estimated to be affecting around one-third of the patients hospitalized with SARS-COV-2 [3,2]. This indicates that a combined prevalence in admitted and non-hospitalized patients may be even more. Similarly, the management of lung fibrosis after COVID-19 infection remains unexplored at large due to a lack of clinical trials. A potential role of antifibrinolytic therapies is suggested based on anecdotal evidence and a proposed similarity in the mechanism of post-COVID-19 lung fibrosis to idiopathic pulmonary fibrosis (IPF) [[2], [3], [4]]. We present a case of successfully treated COVID-19 pneumonia, who went on to develop pulmonary fibrosis.

## Case report

A 60-year-old gentleman, known pre-diabetic, presented with a dry cough for three months. The cough was continuous, subsiding only for short periods during the day with no diurnal pattern. The patient also complained of a progressively increasing dyspnea on minimal exertion. There was no associated fever, weight loss, or night sweats. He was not taking any medication. The patient was a non-smoker and did not consume alcohol.

**Fig. 1:**
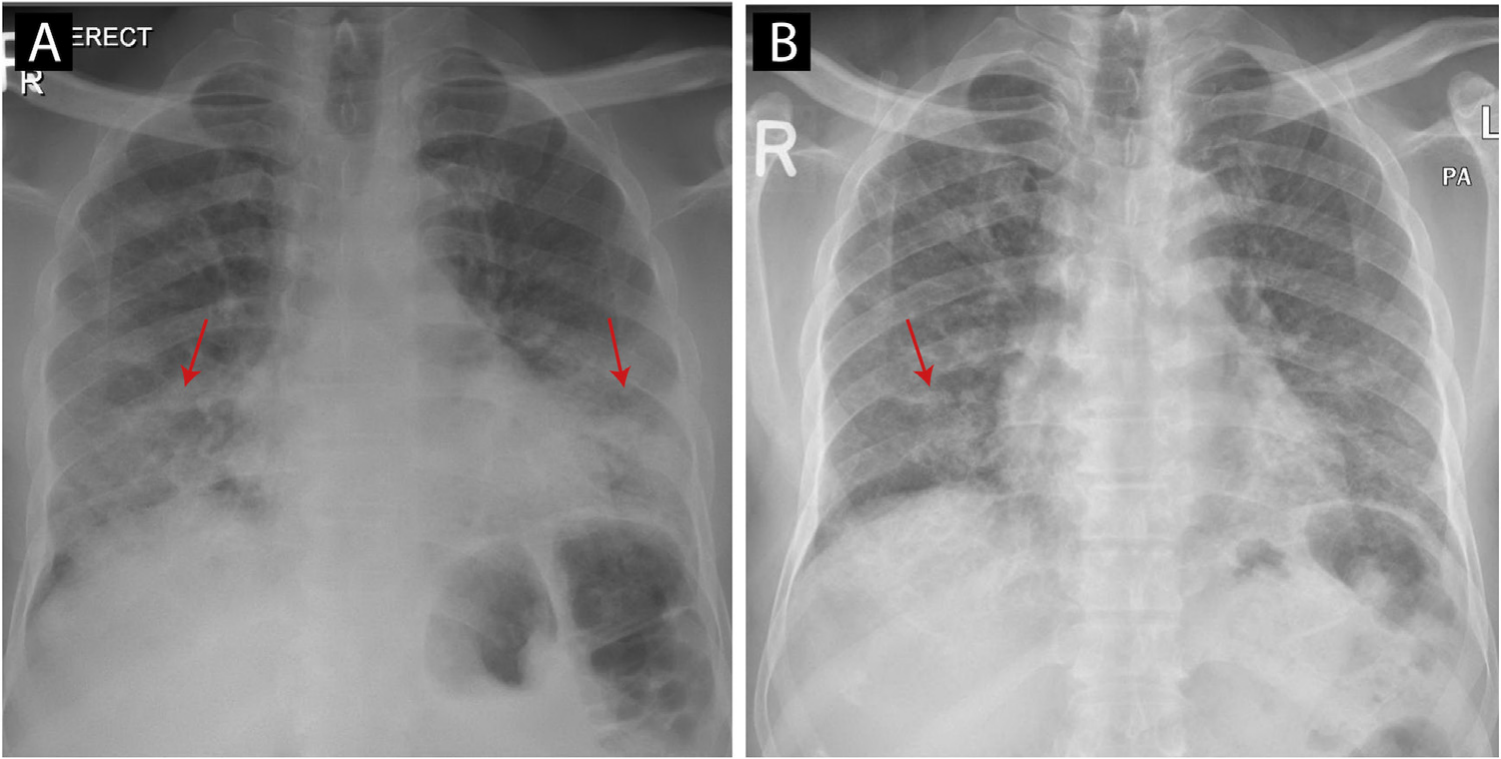
Chest Xray (1a: at the time of COVID-19 infection, discrete areas of airspace consolidation in the bilateral lower and mid zones. 1b: on the second admission, showing resolution of the airspace consolidations in the bilateral lower and mid zones).

He was treated for moderate severity COVID-19 pneumonia four months before his admission. His presentation at that time was with fever, cough. Chest X-ray revealed discrete airspace consolidation areas in the bilateral lower and mid zones [Fig. 1a]. The patient was treated with favipiravir 600 mg twice daily for seven days, intravenous dexamethasone 8 mg daily for ten days, and intravenous amoxicillin-clavulanate 1200 mg twice daily for seven days based on the local COVID-19 infection treatment guidelines at the time. The patient improved on the treatment and was discharged in an asymptomatic condition after ten days. He never required intubation or intensive care unit admission during his hospital stay.

The patient remained symptom-free for around one month before developing the dry cough. The cough progressively worsened over the next three months until he sought medical advice. He was afebrile and hemodynamically stable, saturating normal on room air. Physical examination was significant for bilateral basilar crackles with decreased air entry at the lung bases. The patient had desaturation to 90 % on room air when walking for 10–15 meters. A chest X-ray on this admission showed improvement in the opacities previously seen [Fig. 1b].

The patient’s basic blood profile and biochemistry were normal [Table 1]. Infectious workup was negative for bacterial growth (including *Mycoplasma pneumonia, Legionella pneumophila, and Chlamydia pneumonia*). Nasopharyngeal polymerase chain reaction (PCR) for common respiratory viruses (including Influenza, Parainfluenza, Respiratory syncytial virus, and Middle East respiratory syndrome coronavirus) were negative. A COVID-19 re-infection was considered as a possibility. However, a SARS-CoV-2 nasopharyngeal reverse transcription Polymerase chain reaction (RT-PCR) via the GeneXpert system came back negative. The patient was clinically euvolemic (no limb edema, no rise in jugular venous pressure). Pulmonary tuberculosis was ruled out with negative acid-fast bacilli smears, culture, and PCR (via Xpert MTB nucleic acid amplification).

**Table 1:** Basic labs at admission.

Investigation	Result with normal range
WBC	7.4*10^3/uL (4.0−10.0*10^3/uL)
Hgb	16.4 gm/dL (13−17 gm/dL)
Platelets	229*10^3/uL (150−400*10^3/uL)
Creatinine	70 umol/L (62−106 umol/L)
ALT	28 U/L (0−41 U/L)
AST	22 U/L (0−40 U/L)
CRP	2.8 mg/L (0−5.0 mg/L)
Lactic acid	1.6 mmol/L (0.5−2.2 mmol/L)

**Fig. 2:**
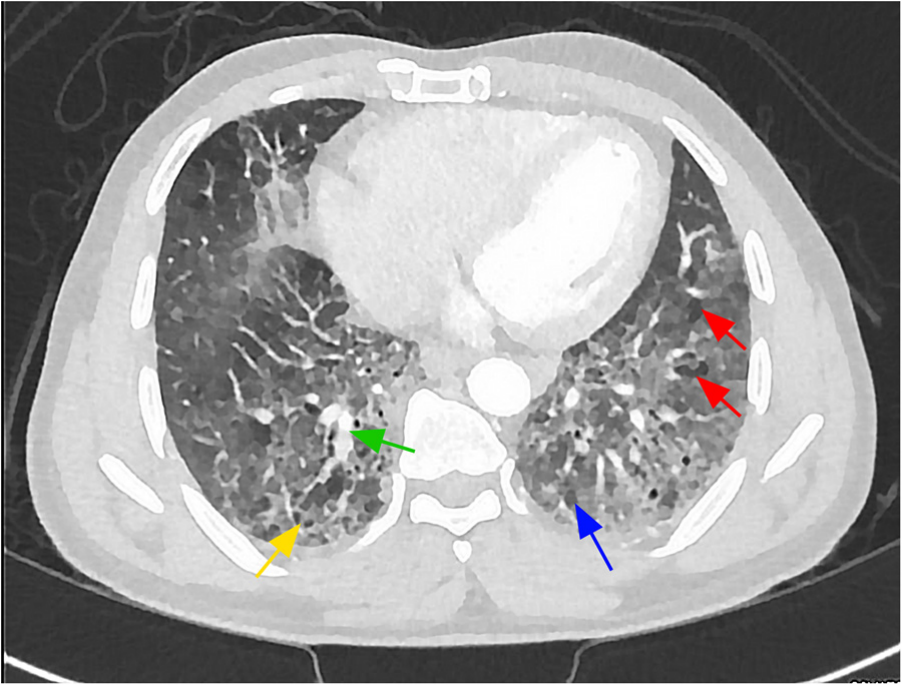
HRCT chest showing bilateral interlobular septal thickening (green arrow), traction bronchiectasis (red arrows), and honeycombing (yellow arrow) with scattered reticular and ground-glass infiltrations (blue arrow), mainly at the lower lung fields and bilateral apex (For interpretation of the references to colour in this figure legend, the reader is referred to the web version of this article).

A high-resolution computerized tomography (HRCT) scan of the chest revealed bilateral interlobular septal thickening, traction bronchiectasis, and honeycombing with scattered reticular and ground-glass infiltrations, mainly at the lower lung fields and bilateral apex (Fig. 2). These findings were suggestive of interstitial pneumonia pattern and early interstitial lung disease. The patient received symptomatic treatment with anti-tussive (dextromethorphan).

The patient was discharged in a stable condition with a resolving cough. He is planned to be seen in the pulmonary clinic for pulmonary function tests and possibly start antifibrinolytic therapy.

## Discussion

The current pandemic of the novel coronavirus SARS‐CoV‐2, causative agent of the COVID-19 infection (coronavirus disease 2019; previously 2019‐nCoV), emerged from China and is currently affecting almost all countries globally [7]. SARS-CoV-2 belongs to the Coronaviruses family, which are enveloped, positive single‐stranded large RNA viruses that infect humans and known for their ability to infect various types of animals [5].

The COVID-19 infection mainly presents with respiratory symptoms inducing a flu-like illness with fever, cough, and asthenia. SARS-CoV-2 can cause severe lung injury. In the high‐risk population like the elderly or those with multiple comorbidities, the virus has a higher prevalence of severe interstitial pneumonia, ARDS, and multi-organ failure. Affected individuals display a variable extent of dyspnea and radiological changes [6,7]. Although the primary target for the SARS-CoV-2 is the pulmonary system, it can involve multiple organ systems such as gastrointestinal, endocrine, and cardiovascular systems [8,9].

Patients affected with pulmonary fibrosis commonly complain of dry cough, fatigue, and dyspnea. Weight loss is expected with physical deconditioning. The deconditioning leads to a decreased functional capacity, a decreased quality of life, and may lead to loss of income. Timely and adequate treatment of COVID-19 infection and other coronaviral diseases does not guarantee prevention from pulmonary fibrosis development later on [10]. SARS-CoV-2 uses angiotensin-2-converting enzyme (ACE2) as a cell receptor in humans, causing interstitial lung damage followed by parenchymal lesions [11].

Although mainly it is considered idiopathic, the inflammatory changes in the lungs secondary to the viral infection can lead to fibrotic changes. This can be more likely with ARDS than pneumonia due to the diffuse alveolar damage and resultant type II pneumocyte hyperplasia [3]. Pulmonary fibrosis is characterized by the lungs’ inability to reconstruct the damaged alveolar epithelium, persistence of fibroblasts, and excessive deposition of collagen and other extracellular matrices (ECM) components. This is accompanied by the destruction and alteration of normal lung architecture [12].

The etiology of pulmonary fibrosis is multifactorial and depends on age, smoking, viral infection, drug exposure, and genetic predisposition [13,14]. Mediators of inflammation, such as Transforming growth factor-beta (TGF-β), vascular endothelial growth factor (VEGF), interleukin 6 (IL-6), and tumor necrosis factor-alpha (TNF-α) play a vital role in the initiation of the fibrotic cascade. Moreover, vascular dysfunction causes the progression of fibrosis [15,16].

One of the hypotheses regarding aggressive disease courses amongst the elderly population is the increased pro-inflammatory cytokines compared to the younger population. The similarity of cytokine profiles in IPF and COVID-19 infection suggests analogous pulmonary fibrosis’s pathophysiology in these diseases [10]. More than a third of recovered patients develop fibrotic abnormalities in radiology. Also, 47 % of patients have an abnormal diffusion capacity of the lungs for carbon monoxide (DLCO), and 25 % have reduced total lung capacity (TLC). This seemed even worse in patients with severe disease [17].

With an already increasing prevalence of pulmonary fibrosis from other causes, post-COVID-19 lung fibrosis poses a considerable morbidity burden considering the number of infections worldwide [18]. Management of post-COVID-19 pulmonary fibrosis is currently an unexplored aspect, and is largely limited to symptomatic management. The role of antifibrotic therapy in treatment as well as prevention of fibrosis post SARS-COV-2 infection is yet to be determined [4].

The current discharge criteria of COVID-19 infection is similar to any other infectious disease. It mainly comprises symptom improvement, laboratory evidence of SARS-CoV-2 viral clearance from the body, and serology (IgG) if available [19]. Although patients at discharge usually maintain saturation at room air, like our patient, there may be more cases with underlying exertional desaturation upon discharge. This should not prevent appropriate discharges if the desaturation is not limiting the daily life activities. However, a 6-minute walk test upon discharge in such patients can give prognostic information on the lungs' underlying subclinical inflammation and possible fibrosis.

## Conclusion

Post-COVID-19 fibrosis is one of the emerging complications of COVID-19 pneumonia and ARDS. It is estimated to be prevalent in around one-third of COVID-19 infected hospitalized patients. More extensive studies are needed to investigate this occurrence and test the efficacy of already tested drugs (for idiopathic pulmonary fibrosis) such as antifibrotics for post−COVID-19 fibrosis. We suggest doing a 6-minute walk test to assess exertional desaturation in patients who are fulfilling other discharge criteria for COVID-19 pneumonia and ARDS.

## Financial support and sponsorship

None

## Consent

Written informed consent was obtained from the patient for publication of this case report and accompanying images

## Author statement

This manuscript is original work and has not been submitted or is not under consideration for publication elsewhere. All the authors have reviewed the manuscript and approved it before submission. None of the authors have any conflict of interest from publishing this work.

## CRediT authorship contribution statement

**Mousa Ahmad Alhiyari:** Conceptualization, Writing - original draft. **Fateen Ata:** Conceptualization, Methodology, Writing - original draft, Writing - review & editing. **Mohd Islam Alghizzawi:** Writing - review & editing. **Ammara Bint I Bilal:** Writing - review & editing. **Ahmad Salih Abdulhadi:** Supervision, Writing - review & editing. **Zohaib Yousaf:** Methodology, Writing - review & editing, Supervision.

## Declaration of Competing Interest

This manuscript is original work and has not been submitted or is not under consideration for publication elsewhere. All the authors have reviewed the manuscript and approved it before submission. None of the authors have any conflict of interest from publishing this work.
